# RETRACTION: Lianhua-Qingwen Displays Antiviral and Anti-Inflammatory Activity and Synergistic Effects with Oseltamivir against Influenza B Virus Infection in the Mouse Model

**DOI:** 10.1155/ecam/9849427

**Published:** 2025-11-26

**Authors:** Evidence-Based Complementary and Alternative Medicine

RETRACTION: C. Yang, Y. Wang, J. He, et al., “Lianhua-Qingwen Displays Antiviral and Anti-Inflammatory Activity and Synergistic Effects with Oseltamivir against Influenza B Virus Infection in the Mouse Model,” *Evidence-Based Complementary and Alternative Medicine*, no. 2020 (2020), https://doi.org/10.1155/2020/3196375.

The above article, published online on 5 June 2020 in Wiley Online Library (https://wileyonlinelibrary.com), has been retracted by John Wiley & Sons Ltd.

The retraction has been agreed following an investigation of the concerns raised by *Actinopolyspora biskrensis* on PubPeer [1], which identified multiple instances of overlapping figures as follows:

- Figure 5: The images of the 100 and 200 mg/kg/day LQ panels are identical.
- Figure 7: The image of the uninfected tissue in panel a contains overlapping features with panel e, which depicts tissue belonging to a different treatment group.

The authors responded to these concerns and provided raw data; however, concerns regarding the reliability of the information presented in the manuscript remain and it is therefore retracted.

The authors agree with this retraction.
